# Incidence and Risk Factors of in-hospital mortality from AKI after non-cardiovascular operation: A nationwide Survey in China

**DOI:** 10.1038/s41598-017-13763-9

**Published:** 2017-10-24

**Authors:** Yu Pan, Wenji Wang, Jinwei Wang, Li Yang, Feng Ding, ISN AKF 0by25 China Consortium, Minjie Zhou, Minghui Zhao, Hanyan Wang, Guolan Xing, Zhangsuo Liu, Li Wang, Fang Wang, Yonggui Wu, Dan Huang, Suhua Li, Shu Wang, Gang Xu, Shuwang Ge, Qiang He, Quanquan Shen, Jianghua Chen, Ping Zhang, Menghua Chen, Lihua Wu, Xiaohua Liu, Miao Pan, Zaizhi Zhu, Qianting Zou, Lin Yang, Ping Zhu, Xiyan Lian, Jintao Zhao, Yun Li, Lin Yang, Huamin Wang, Wenping Hu, Jianqin Wang, Jing Wang, Rong Wang, Bing Liu, Changlin Mei, Tong Zhang, Jixian Xu, Jianxin Han, Rongshan Li, Tao Wen, Juan Cao, Liang Zhang, Yan Wang, Jinhua Xu, Beiyan Bao, Bicheng Liu, Hongyu Chen, Shaomei Li, Yan Zha, Qiong Luo, Dongchen Chen, Yulan Shen, Yunhua Liao, Zhengrong Zhang, Xianqiu Wang, Kun Zhang, Luojin Liu, Peiju Mao, Chunxiang Guo, Jiangang Li, Zhenfu Wang, Shoujun Bai, Shuangjie Shi

**Affiliations:** 1 Division of Nephrology, Shanghai Ninth People’s Hospital, School of Medicine, Shanghai Jiaotong University, Shanghai, 200011 P.R. China; 2 Division of Nephrology, Department of Medicine, Peking University First Hospital; Peking University Institute of Nephrology; Key Laboratory of Renal Disease, Ministry of Health of China, Beijing, 100034 P.R. China; 3 The First Affiliated Hospital of Zhengzhou University, Zhengzhou, China; 4 Sichuan Provincial People’s Hospital, Chengdu, China; 5 The First Affiliated Hospital of Anhui Medical University, Anhui, Hefei China; 6 The First Affiliated Hospital of Xinjiang Medical University, Urumqi, China; 7 Tongji Hospital, Tongji Medical College, Huazhong University of Science and Technology, Wuhan, China; 8 Zhejiang Provincial People’s Hospital, Hangzhou, China; 9 The First Affiliated Hospital of Zhejiang University, Hangzhou, China; 10 General Hospital of Ningxia Medical University, Yinchuan, China; 11 Ningde Municipal Hospital, Fujian Medical University, Ningde, China; 12 Meishan City People’s Hospital, Meishan, China; 13 The First College of Clinical Medical Science, China Three Gorges University, Yichang, China; 14 The Second Affiliated Hospital of Kunming Medical University, Kunming, China; 15 Jiangxi Provincial People’s Hospital, Nanchang, China; 16 The Fourth Affiliated Hospital of Harbin Medical University, Harbin, China; 17 Lanzhou University Second Hospital, Lanzhou, China; 18 Shandong Provincial Hospital Affiliated to Shandong University, Jinan, China; 19 Shanghai Changzheng Hospital, The Second Military Medical University, Shanghai, China; 20 Renshou County People’s Hospital, Renshou, China; 21 The Affiliated Provincial People’s Hospital of Shanxi Medical University, Taiyuan, China; 22 Taixing People’s Hospital, Taixing, China; 23 Ordos Central Hospital, Ordos, Inner Mongolia China; 24 Xinganmeng People’s Hospital, Wulanhaote, Inner Mongolia China; 25 Fuyang City People’s Hospital, Fuyang, Zhejiang China; 26 Ningbo Yinzhou Second Hospital, Ningbo, China; 27 Zhongda Hospital, Southeast University, Nanjing, China; 28 Hangzhou Hospital of Traditional Chinese Medicine, Hangzhou, China; 29 The Second Hospital of Hebei Medical University, Shijiazhuang, China; 30 Guizhou Provincial People’s Hospital, Guizhou Medical University, Guiyang, China; 31 Peking University Shenzhen Hospital, Shenzhen, China; 32 Hengxian People’s Hospital, Hengxian, China; 33 Miyun County Hospital, Beijing, China; 34 The First Affiliated Hospital of Guangxi Medical University, Nanning, China; 35 Puer City People’s Hospital, Puer, China; 36 Zoucheng City People’s Hospital, Zoucheng, China; 37 Taihe Hospital of Traditional Chinese Medicine, Taihe, China; 38 Shenzhen Longhua New District Central Hospital, Shenzhen, China; 39 Tongren Hospital Shanghai Jiao Tong University School of Medicine, Shanghai, China; 40 Zhongwei City People’s Hospital, Zhongwei, China; 41 Huaxian People’s Hospital, Huaxian, China; 42 Suihua City First Hospital, Suihua, China; 43 Qingpu Branch of Zhongshan Hospital, Fudan University, Shanghai, China; 44 Qingxuxian City People’s Hospital, Qingxu, China

## Abstract

This study aimed to describe the mortality and risk factors of in-hospital mortality from acute kidney injury (AKI) after non-cardiovascular operation in China based on a nationwide survey about AKI. The study sample was drawn from ISN AKF 0by25 China Consortiums, which is a nationwide, cross-sectional survey from 22 provinces in mainland China. AKI after non-cardiovascular operation was identified according to the 2012 KDIGO AKI creatinine criteria or expanded criteria. In total, 3468 cases were identified as hospital-acquired AKI (HA-AKI). Of these, 1059 cases were defined as AKI after major non-cardiovascular surgery. Post-operative AKI and non-operative AKI were similar in aetiology and in the need for RRT intervention. The all-cause in-hospital mortality was 17.0% (180 of 1059) among patients with AKI after a major surgery. Older age (OR = 1.14, p = 0.046), more severe comorbidities (OR = 9.29, p < 0.001), a history of CVD (OR = 1.85, p = 0.007), more severe peak AKI stage, and being located in the northwest region of China (OR = 2.47, p = 0.012) were all significantly associated with increased in-hospital mortality risk in AKI patients who underwent an operation. AKI after a non-cardiovascular operation has become a huge medical burden in China. The features of operative AKI varied substantially in different regions of China. Increased attention must be paid to the occurrence of potential intrinsic renal AKI when patients are exposed to nephrotoxic factors or comorbidities.

## Introduction

Acute kidney injury (AKI) is associated with high mortality and morbidity worldwide^1–3^. In 2013, to improve the diagnosis and treatment of AKI worldwide, the International Society of Nephrology (ISN) launched the “0 by 25” global target, that is, zero deaths in patients with untreated acute kidney failure by 2025^4^. As part of the global initiative, the ISN Acute Kidney Failure 0by25 China Consortium conducted a nationwide survey in the hospitalized population and reported a substantial burden of AKI in China^5^.

AKI after major, non-cardiovascular surgery was independently associated with significant morbidity and in-hospital mortality^6–8^. In addition, post-operative AKI has been associated with longer-term adverse events including the development of chronic kidney disease (CKD) and late mortality^9^. AKI after non-cardiovascular surgery occurs in approximately 7.1% to 39% of all surgery cases^7,10,11^. However, a causative role of AKI associated with major non-cardiovascular surgery is not yet fully recognized^6^. Until now, data about the incidence and risk factors of AKI after major surgery in China have been sparse.

We designed the China national survey of AKI for adults treated in-hospital in 2013 to provide reliable data to estimate the characteristics, death risk factors, and prognosis of acute kidney injury (AKI) in patients undergoing major non-cardiac and vascular surgery in various regions.

## Method

### Study design and participants

The study sample was drawn from ISN AKF 0by25 China Consortiums, which is a nationwide, cross-sectional survey of adult patients who were admitted in-hospital in 2013 in academic or local hospitals from 22 provinces in mainland China^5^. In brief, we included 22 of the 31 provinces, municipalities, and autonomous regions in China in our survey, covering 82% of the country’s population and the four geographical regions of China (north, northwest, southeast, and southwest). We enrolled an academic hospital in each region’s capital city and a local hospital from a smaller city or rural county. This study was approved by the ethics committees of Peking University First Hospital and the enrolled study hospitals. All experimental protocols were performed in accordance with relevant guidelines and regulations. Written informed consent was obtained from either the patients’ legal guardians or themselves prior to participation, in accordance with the Declaration of Helsinki.

### Survey design

The AKI definition from the 2012 Kidney Disease: Improving Global Outcomes (KDIGO) was used as the major screening criteria^12^. For patients who had repeated serum creatinine assays with intervals longer than 7 days and those who had recovering AKI, we expanded the screening criteria to a serum creatinine increase or decrease of 26.5 μmol/L or more during hospital stay.

Patients who had chronic kidney disease stage 5, nephrectomy, kidney transplantation, or a peak serum creatinine level of less than 53 μmol/L were excluded. Patients who met the identification criteria but whose serum creatinine change could not be attributed to AKI (e.g., a serum creatinine decrease after amputation) were also excluded. Patients who had undergone cardiac surgery, abdominal aortic surgery, angiography and/or stenting procedure and urological surgery were also excluded.

For patients who were confirmed as having AKI by our survey (detected AKI), investigating records were completed to document the sociodemographic status, comorbidities, clinical departments, diseases or conditions that could cause renal hypoperfusion or urinary obstruction, nephrotoxic drugs and environmental nephrotoxins, invasive procedures and surgeries, critical illness, AKI classification, renal replacement therapy (RRT), renal referral, length of hospital stay, and all-cause in-hospital death. AKI staging (1 to 3) was performed at three time points: when AKI was diagnosed, when AKI was at its peak (i.e., the highest AKI stage that a patient reached during their entire in-hospital stay), and when AKI was recognized by the physicians in charge. We defined renal recovery at discharge as full recovery with serum creatinine reduced to below the threshold level or to the baseline. We defined partial recovery as serum creatinine reduced by 25% or more from peak concentration but remaining higher than the threshold or baseline. We defined failure to recover as a patient still dependent on dialysis or serum creatinine reduced by less than 25% from peak concentration.

### Statistical analysis

We presented the continuous data as the mean (SD) or median (IQR) as appropriate and the categorical variables as n (%). We described the characteristics of the patients and the statues of recognition and treatment of AKI, stratified by hospital level, geographical region, and level of economic development. We compared groups using a one-way ANOVA or the Kruskal-Wallis test for continuous variables and the χ² test for categorical variables.

We analysed relevant covariates that might be associated with the in-hospital mortality of AKI (yes vs no) using a multivariable logistic regression and reported odds ratios with 95% CIs and p values from the Wald χ² test. The covariates included in the analysis were age (change by 10 years), sex (male vs female), chronic kidney disease (yes vs no), renal referral (yes vs no), AKI stages at detection and at peak, hospital levels (academic vs local), economic development (by tertiles of gross domestic product per head), and geographic regions.

We used EpiData software (version 3.1, EpiData Association, Odense, Denmark) for data entry and management. All p values are two-sided, and a p value of less than 0.05 was deemed significant. The analyses were performed with SAS software (version 9.1, SAS Institute, Cary, NC, USA).

## Results

### Overview of AKI after Major non-cardiovascular Surgical Cases

**Figure 1:**
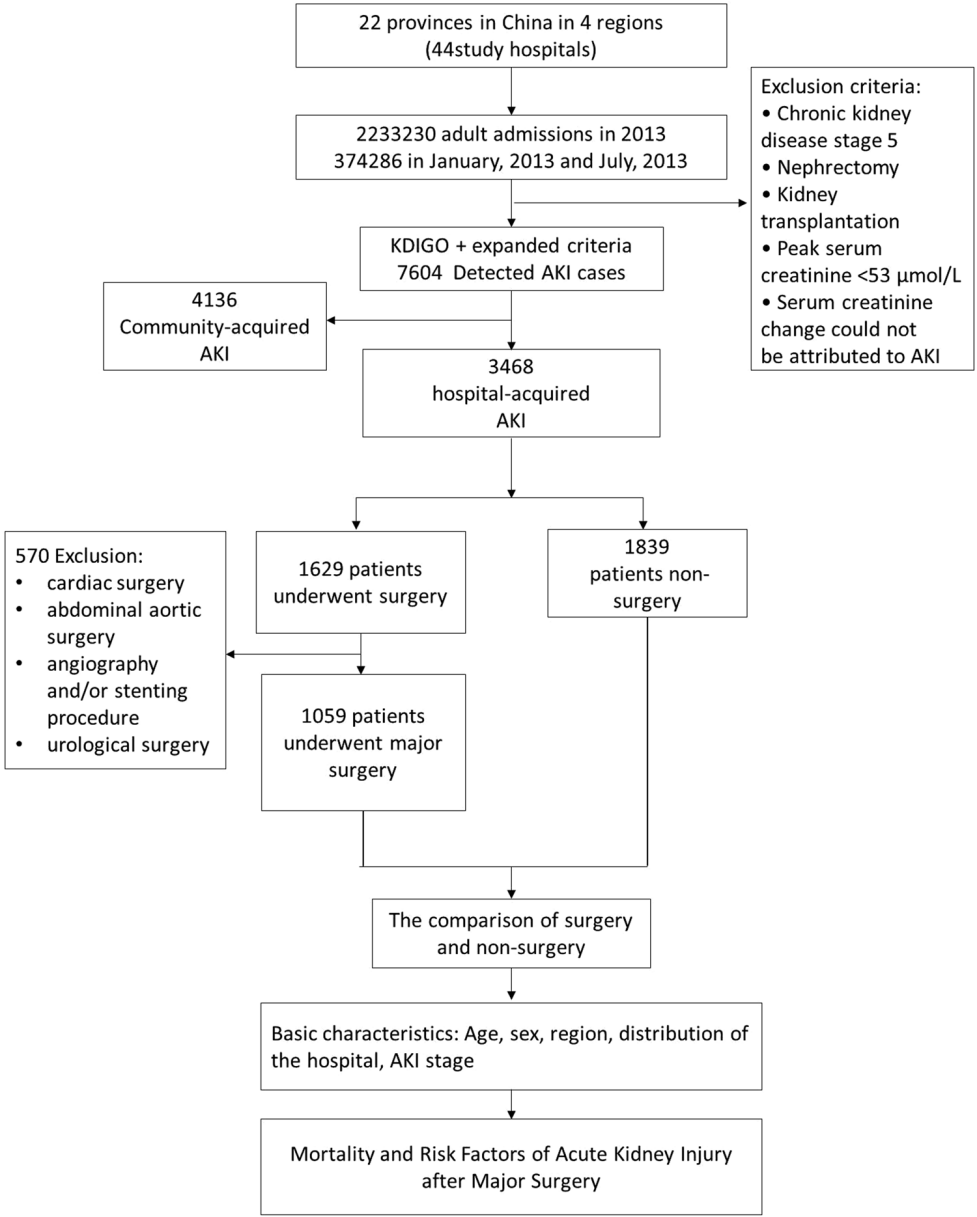
Study profile.

Among the 374,286 patients who were admitted to the hospital during either of the 2 months studied (January and July of 2013), AKI was defined in 7604 cases, of which 3468 were identified as hospital-acquired AKI (HA-AKI). Of this group, 1629 were identified as AKI after surgery. The detection rate of AKI after surgery among adult hospitalizations was 0.4% (1629 of 374,286 cases), and the proportion of AKI after surgery among all AKI cases was 47% (1629 of 3468 cases), of which AKI after major non-cardiovascular surgery occurred in 1059 cases (Fig. 1).

### Characteristics of AKI with and without major non-cardiovascular Surgery

We assessed 2898 patients with AKI in this survey: 1059 patients undergoing a major non-cardiovascular operation and 1839 non-surgical patients. The characteristics of the patients are shown in Table 1. Approximately 58% of the patients detected with AKI were older than 60 years. Compared to non-surgical patients, the patients who underwent operations were younger (64.1 ± 17.5 vs 59.3 ± 16.5 years, p < 0.001, respectively) and had less medical history, such as cardiovascular disease, hypertension, diabetes and chronic kidney disease. However, the patients who underwent operations had a higher incidence of malignancy compared to non-surgical patients (30.9% vs. 21.8%, p < 0.001, respectively). Pre-renal AKI occurred more frequently in patients who underwent an operation (60.8%) than in non-surgical patients (53.1%, p < 0.0001). Intrinsic-renal AKI occurred more frequently in non-surgical patients (29.4%) than in patients who underwent an operation (24.0%, p < 0.0001). The most common injury factors that might be implicated in the development of AKI included renal hypoperfusion and nephrotoxic drugs or environmental nephrotoxins; these factors were common in both AKI patients. Compared to patients who did not undergo an operation, patients who underwent an operation showed a higher proportion of exposure to nephrotoxic drugs such as aminoglycosides (3.0% vs 6.1%, respectively, p < 0.001), vancomycin (5.1% vs. 7.7%, respectively, p = 0.004), and other antibiotics (45.6% vs. 57.6%, respectively, p < 0.001). More than half of the non-surgical patients had stage 1 AKI (52.6%), whereas more than half of the patients who underwent an operation had stage 2 (28.1%) and 3 (24.0%) AKI. The existence of complicated comorbidities was also slightly different between those who did and did not undergo an operation. The most common comorbidity of surgical patients was shock, followed by multiple organ dysfunction syndrome (MODS); however, MODS was the primary comorbidity in non-surgical patients. Compared to non-surgical patients, renal referral was performed less frequently in patients with an operation (18.9% vs. 13.6%, p < 0.001, respectively), but there was no significant difference in the RRT/RRT ratio. Although AKI after major surgery has a relatively mild outcome, 66% of the patients with renal function recovered. Meanwhile, a higher non-recognition rate of AKI by the physicians was recorded in the patients who underwent an operation. The patients who underwent an operation had a longer hospital stay than the non-surgical patients (22 vs. 19 days, p < 0.001). The above data show that compared to the non-surgical AKI patients, the AKI patients who underwent an operation had higher proportions of nephrotoxic drug exposure, the comorbidity of shock and a malignancy history. There were no significant differences in primary aetiologies (hypoperfusion, nephrotoxicity and sepsis) between post-operative AKI and non-operative AKI.

**Table 1:** Characteristics of patients with AKI with or without operations.

	Total	Without operations	With operations	*P* value
**Number of patients Demographics**	2898	1839	1059	
Age group, years (x ± SD)	62.3 ± 17.3	64.1 ± 17.5	59.3 ± 16.5	<0.001
Aged 18–39	308 (10.6%)	183 (10.0%)	125 (11.8%)	<0.001
Aged 40–59	889 (30.7%)	510 (27.7%)	379 (35.8%)	
Aged 60–79	1159 (40.0%)	728 (39.6%)	431 (40.7%)	
Aged ≥80	542 (18.7%)	418 (22.7%)	124 (11.7%)	
Male, n (%)	1884 (65.0%)	1173 (63.8%)	711 (67.1%)	0.068
**Region**				
North	791 (27.3%)	509 (27.7%)	282 (26.6%)	0.088
Northwest	1333 (46.0%)	824 (44.8%)	509 (48.1%)	
Southeast	280 (9.7%)	171 (9.3%)	109 (10.3%)	
Southwest	494 (17.0%)	335 (18.2%)	159 (15.0%)	
**Medical history, n (%)**				
Previous CVD	992 (34.2%)	756 (41.1%)	236 (22.3%)	<0.001
Previous HT	1232 (42.5%)	846 (46.0%)	386 (36.5%)	<0.001
Previous DM	584 (20.2%)	415 (22.6%)	169 (16.0%)	<0.001
Pre-exsisting CKD	684 (23.6%)	512 (27.8%)	172 (16.2%)	<0.001
Malignancy	727 (25.1%)	400 (21.8%)	327 (30.9%)	<0.001
**Cause of AKI, n (%)**				
Pre-renal	1621 (55.9%)	977 (53.1%)	644 (60.8%)	<0.001
Intrinsic-renal	795 (27.4%)	541 (29.4%)	254 (24.0%)	0.002
Post-renal	133 (4.59%)	87 (4.73%)	46 (4.34%)	0.632
Unclassified	349 (12.0%)	234 (12.7%)	115 (10.9%)	0.137
**Injury factors, n(%)**				
Hypoperfusion	2319 (80.0%)	1463 (79.6%)	856 (80.8%)	0.408
Nephrotoxicity	2444 (84.3%)	1543 (83.9%)	901 (85.1%)	0.402
Sepsis	225 (7.8%)	138 (7.5%)	87 (8.2%)	0.491
Others	1529 (52.8%)	877 (47.7%)	652 (61.6%)	<0.001
**Drugs, n (%)**				
Aminoglycosides	120 (4.1%)	55 (3.0%)	65 (6.1%)	<0.001
Vancomycin	176 (6.1%)	94 (5.1%)	82 (7.7%)	0.004
Antifungal drugs	298 (10.3%)	202 (11.0%)	96 (9.1%)	0.101
Antiviral durgs	177 (6.1%)	129 (7.0%)	48 (4.5%)	0.007
Antibiotics	1448 (50.0%)	838 (45.6%)	610 (57.6%)	<0.001
NSAIDs	423 (14.6%)	248 (13.5%)	175 (16.5%)	0.0256
Immunosuppressive agents	154 (5.31%)	79 (4.3%)	75 (7.1%)	<0.001
Chemotherapy drugs	176 (6.1%)	122 (6.6%)	54 (5.1%)	0.096
Chinese herbal medicine	49 (1.7%)	36 (2.0%)	13 (1.2%)	0.142
others	1910 (65.9%)	1266 (68.8%)	644 (60.8%)	<0.001
**AKI stage, n(%)**				
1	1474 (50.9%)	967 (52.6%)	507 (47.9%)	0.032
2	746 (25.7%)	448 (24.4%)	298 (28.1%)	
3	678 (23.4%)	424 (23.1%)	254 (24.0%)	
**Comorbidities, n(%)**				
MODS	471 (16.3%)	323 (17.6%)	148 (14.0%)	0.012
ARDS	224 (7.7%)	141 (7.7%)	83 (7.8%)	0.869
SEPSIS	225 (7.8%)	138 (7.5%)	87 (8.2%)	0.491
SHOCK	414 (14.3%)	240 (13.1%)	174 (16.4%)	0.012
DIC	47 (1.62%)	24 (1.31%)	23 (2.17%)	0.075
**Treatment of AKI, n(%)**				
Specialist consultant				
Renal referral	492 (17.0%)	348 (18.9%)	144 (13.6%)	<0.001
RRT	169 (5.8%)	111 (6.0%)	58 (5.5%)	0.536
RRT indication	316 (10.9%)	211 (11.5%)	105 (9.9%)	0.195
**Hospital stay, (day)**	20 (11–34)	19 (10–32)	22 (13–36)	<0.001
In-hospital mortality, n (%)*	572 (20.1%)	392 (21.7%)	180 (17.4%)	0.006
**Renal prognosis, n (%)**				
Recovery	1717 (59.3%)	1015 (55.2%)	702 (66.3%)	<0.001
Recurred*	138 (4.9%)	81 (4.5%)	57 (5.6%)	0.206
Maintenance RRT*	60 (47.2%)	44 (50.0%)	16 (41.0%)	0.350
Diagnosis*				0.018
Omission diagnosis, n (%)	2335 (80.9%)	1461 (79.7%)	874 (83.0%)	
Delayed diagnosis, n (%)	139 (4.8%)	83 (4.5%)	56 (5.3%)	
Immediate diagnosis, n (%)	412 (14.3%)	289 (15.8%)	123 (11.7%)	

*Data missing for in-hospital mortality in 55 cases (31 in AKI patients without operations vs 24 in AKI patients with operations), for recurred renal prognosis in 99 cases (55 vs 44), for maintenance RRT of renal prognosis in 2771 cases (1751 vs 1020), and for diagnosis in 12 cases (6 vs 6). CVD: Cardiovascular disease; HT: hypertension; DM: diabetes mellitus; CKD: chronic kidney disease; NSAIDs: nonsteroidal anti-inflammatory drugs; MODS: multiple organ dysfunction syndrome; ARDS: Acute respiratory distress syndrome; DIC: disseminated intravascular coagulation; RRT: renal replacement therapy.

### Features and causes of AKI after major non-cardiovascular Surgery

When examined in strata defined by regions, decreased kidney perfusion-induced AKI was predominant in northwest China (77.1%), but it accounted for 56.0%, 63.1%, and 50.9% of AKI cases in north, southeast and southwest China, respectively (p < 0.001). AKI stage 3 was also more common in northwest China (30.3%) than in other regions of China (p = 0.04); however, the rate of previous CVD history was lower in the northwest than in the north, southeast and southwest (12.8% vs. 28.4%, 20.8%, and 22.6%, p = 0.006, respectively). Although there were no significant differences between areas regarding renal referral and RRT treatment/RRT indication rate, higher in-hospital mortalities were observed in northwest China (20.4%) and southeast China (21.9%) (Table 2). In addition, the patients in the northwest (11.9%) and southwest (15.7%) had a lower rate of intrinsic-renal AKI than the patients in north (35.1%) and southeast (23.0%) China. Northwest China had a higher non-recognition rate of AKI (94/109, 86.2%) and a lower immediate diagnosis rate (10/109, 9.2%).

**Table 2:** Characteristics of patients with postoperative AKI according to regions.

	Total	North	Southeast	Northwest	Southwest	*P* value
**Number of patients**	1059	282	509	109	159	
**Demographics**						
Age group, years (x ± SD)	59.3 ± 16.5	57.7 ± 17.2	59.2 ± 16.0	58.6 ± 16.6	63.1 ± 16.6	0.013
Male, n (%)	711 (67.1%)	187 (66.3%)	336 (66.0%)	83 (76.2%)	105 (66.0%)	0.214
**Medical history, n(%)**						
Previous CVD	236 (22.3%)	80 (28.4%)	106 (20.8%)	14 (12.8%)	36 (22.6%)	0.006
Previous HT	386 (36.5%)	116 (41.1%)	177 (34.8%)	36 (33.0%)	57 (35.9%)	0.276
Previous DM	169 (16.0%)	50 (17.7%)	67 (13.2%)	16 (14.7%)	36 (22.6%)	0.029
Pre-exsisting CKD	172 (16.2%)	54 (19.2%)	73 (14.3%)	12 (11.0%)	33 (20.8%)	0.053
Malignancy	327 (30.9%)	97 (34.4%)	163 (32.0%)	28 (25.7%)	39 (24.5%)	0.097
**Type of operations**						
Abdominal	366 (34.6%)	90 (31.9%)	188 (36.9%)	31 (28.4%)	57 (35.9%)	0.255
Bone/injury	128 (12.1%)	16 (5.7%)	66 (13.0%)	18 (16.5%)	28 (17.6%)	<0.001
Thoracic	82 (7.7%)	15 (5.3%)	43 (8.5%)	11 (10.1%)	13 (8.2%)	0.314
neurosurgery	178 (16.8%)	48 (17.0%)	90 (17.7%)	20 (18.4%)	20 (12.6%)	0.474
others	277 (26.2%)	92 (32.6%)	112 (22.0%)	23 (21.0%)	50 (31.5%)	0.002
**Cause of AKI, n(%)**						
Pre-renal	644 (60.8%)	158 (56.0%)	321 (63.1%)	84 (77.1%)	81 (50.9%)	<0.001
Intrinsic-renal	254 (24.0%)	99 (35.1%)	117 (23.0%)	13 (11.9%)	25 (15.7%)	<0.001
Post-renal	46 (4.3%)	13 (4.6%)	27 (5.3%)	0 (0.0%)	6 (3.8%)	0.049
Unclassified	115 (10.9%)	12 (4.3%)	44 (8.6%)	12 (11.0%)	47 (29.6%)	<0.001
**AKI stage, n(%)**						0.040
1	507 (47.9%)	141 (50.0%)	238 (46.8%)	42 (38.5%)	86 (54.1%)	
2	298 (28.1%)	69 (24.5%)	146 (28.7%)	34 (31.2%)	49 (30.8%)	
3	254 (24.0%)	72 (25.5%)	125 (24.6%)	33 (30.3%)	24 (15.1%)	
**Treatment of AKI**, n(%)						
Specialist consultant						
Renal referral	144 (13.6%)	33 (11.7%)	68 (13.4%)	17 (15.6%)	26 (16.4%)	0.516
RRT	58 (5.5%)	16 (5.7%)	27 (5.3%)	6 (5.5%)	9 (5.7%)	0.996
RRT indication	105 (9.9%)	26 (9.2%)	49 (9.6%)	6 (5.5%)	24 (15.1%)	0.062
**Hospital stay**	22 (13–36)	27 (14–48)	20 (13–33)	22 (14–34)	21 (11–36)	0.002
In-hospital mortality, n(%)*	180 (17.4%)	48 (17.1%)	76 (15.5%)	22 (20.4%)	34 (21.9%)	0.244
**Renal prognosis,n(%)**						
Recovery	702 (66.3%)	183 (64.9%)	333 (65.4%)	81 (74.3%)	105 (66.0%)	0.313
Recurred*	57 (5.6%)	25 (9.4%)	24 (4.9%)	3 (3.0%)	5 (3.1%)	0.014
Maintenance RRT*	16 (41.0%)	4 (28.6%)	7 (43.8%)	0 (0.0%)	5 (62.5%)	0.341
Diagnosis*						0.429
**Omission diagnosis, n(%)**	874 (83.0%)	239 (85.4%)	413 (81.5%)	94 (86.2%)	128 (81.5%)	
**Delayed diagnosis, n(%)**	56 (5.3%)	10 (3.6%)	30 (5.9%)	5 (4.6%)	11 (7.0%)	
**Immediate diagnosis, n(%)**	123 (11.7%)	31 (11.1%)	64 (12.6%)	10 (9.2%)	18 (11.5%)	

*Data missing for in-hospital mortality in 24 cases (1 in north vs 18 in northwest vs 1 in southeast vs 4 in southwest), for recurred renal prognosis in 44 cases (15 vs 19 vs 10 vs 0), for maintenance RRT of renal prognosis in 1020 cases (268 vs 493 vs 108 vs 151), and for diagnosis in 6 cases (2 vs 2 vs 0 vs 2).

We recorded a very high non-recognition rate of AKI after operation. Compared to non-surgical patients, non-recognition of AKI after operation was predominant in patients who were younger (ages 40 to 79 years), had a higher rate of kidney hypoperfusion and had a lower rate of intrinsic renal injury (Table 3). We further examined strata defined by the recognition of AKI with operation during hospital stay (Table 4). Compared to the timely recognition of AKI, those with non-recognized AKI had a lower per capita GDP (27.8%, p = 0.002), a higher rate of renal hypoperfusion (62.4%, p = 0.01), more like to non-CKD basis (86.7%, p < 0.001) and a milder AKI stage (54.5%, p < 0.001).

**Table 3:** Characteristics of AKI patients with omission diagnosis.

	Total	Without operations	With operations	*P* value
**Number of patients**	2335	1461	874	
Aged 18–39	241 (10.3%)	144 (9.9%)	97 (11.1%)	<0.001
Aged 40–59	729 (31.2%)	412 (28.2%)	317 (36.3%)	
Aged 60–79	942 (40.3%)	573 (39.2%)	369 (42.2%)	
Aged ≥ 80	423 (18.1%)	332 (22.7%)	91 (10.4%)	
**Cause of AKI, n(%)**				
Pre-renal	1338 (57.3%)	793 (54.3%)	545 (62.4%)	<0.001
Intrinsic-renal	576 (24.7%)	388 (26.6%)	188 (21.5%)	0.006
Post-renal	110 (4.7%)	75 (5.1%)	35 (4.0%)	0.213
Unclassified	311 (13.3%)	205 (14.0%)	106 (12.1%)	0.190
**AKI stage, n(%)**				0.109
1	1331 (57.0%)	855 (58.5%)	476 (54.5%)	
2	599 (25.7%)	355 (24.3%)	244 (27.9%)	
3	405 (17.3%)	251 (17.2%)	154 (17.6%)

**Table 4:** Recognition of AKI after major operations by physicians in charge during hospital stay.

	Total	Non-recognised AKI	Delayed recognition of AKI	Timely recognition of AKI	*P* value
**Number of patients**	1053*	874	56	123	0.003
Aged 18–39		97 (11.1%)	5 (8.9%)	23 (18.7%)	
Aged 40–59		317 (36.3%)	17 (30.4%)	42 (34.1%)	
Aged 60–79		369 (42.2%)	20 (35.7%)	40 (32.5%)	
Aged ≥ 80		91 (10.4%)	14 (25.0%)	18 (14.6%)	
**Hospital level**					0.050
Academic hospitals		741(84.8%)	42 (75.0%)	97 (78.9%)	
Local hospitals		133 (15.2%)	14 (25.0%)	26(21.1%)	
**GDP per head**					0.002
Tertile 1		243 (27.8%)	16 (28.6%)	24 (19.5%)	
Tertile 2		355(40.6%)	14 (25.0%)	42 (34.1%)	
Tertile 3		276 (31.6%)	26 (46.4%)	57 (46.3%)	
**CKD factors**					<0.001
Non-CKD		758(86.7%)	42 (75.0%)	82 (66.7%)	
CKD basis		116 (13.3%)	14 (25.0%)	41 (33.3%)	
**Cause of AKI, n** (**%)**					0.012
Pre-renal		545 (62.4%)	28 (50.0%)	68 (55.3%)	
Intrinsic-renal		188 (21.5%)	20 (35.7%)	44 (35.8%)	
Post-renal		35 (4.0%)	5 (8.9%)	6 (4.9%)	
Unclassified		106 (12.1%)	3 (5.4%)	5 (4.1%)	
**AKI stage, n** (**%)**					<0.001
1		476 (54.5%)	7 (12.5%)	23 (18.7%)	
2		244 (27.9%)	17 (30.4%)	36 (29.3%)	
3		154 (17.6%)	32 (57.1%)	64 (52.0%)	

*Data missing for loss diagnosis (n = 6).

### Clinical outcomes in AKI patients with and without major surgery

The all-cause in-hospital mortality was 17.0% (180 of 1059) among patients with AKI after major surgery. In total, 66.3% (702 of 1059) reached complete recovery of kidney function. The multivariable analysis revealed that older age (OR = 1.14, p = 0.046), more severe comorbidities (OR = 9.29, p < 0.001), a history of CVD (OR = 1.85, p = 0.007) and more severe peak AKI stage were associated with a higher in-hospital mortality. Notably, male sex (OR = 1.38, p = 0.026) and RRT indication (OR = 2.22, p < 0.001) were associated with higher in-hospital mortality, but the southeast region (OR = 0.50, p = 0.001) and a nephrology referral (OR = 0.50, p < 0.001) lowered the in-hospital mortality risk in patients who underwent an operation but not in non-surgical patients. Furthermore, compared to other regions of China, the northwest (OR = 2.47, p = 0.012) was significantly associated with increased in-hospital mortality risk in AKI patients who underwent an operation, but non-recognition of AKI may be an independent protective factor (OR = 0.40, p = 0.002) (Table 5). Therefore, there were subtle differences in the risk factors of in-hospital mortality between surgical and non-surgical AKI patients.

**Table 5:** Multivariable logistic regression analysis for factors associated with in-hospital mortality in AKI patients with or without operations.

variables	Total AKI		AKI Without operations		AKI With operations	
	OR (95% CI)	*P* value	OR (95% CI)	*P* value	OR (95% CI)	*P* value
Age (per 10 years increase)	1.27 (1.19–1.37)	<0.001	1.35 (1.23–1.48)	<0.001	1.14 (1.00–1.29)	0.046
Sex (male vs female)	1.34 (1.07–1.69)	0.013	1.38 (1.04–1.82)	0.026	1.24 (0.81–1.88),	0.324
History of CVD (yes or no)	1.25 (0.98–1.58)	0.075	0.99 (0.74–1.33)	0.949	1.85 (1.18–2.90)	0.007
Diabetes (yes or no)	1.10(0.84–1.42)	0.496	1.24 (0.91–1.68)	0.168	0.76 (0.45–1.30)	0.321
CKD (yes or no)	1.03 (0.79–1.34)	0.845	0.93 (0.67–1.28)	0.635	1.38 (0.83–2.30)	0.215
**Diagnosis**						
Immediate diagnosis	Reference		Reference		Reference	
Omission diagnosis	0.76 (0.55–1.04)	0.090	1.06 (0.72–1.57)	0.766	0.40 (0.23–0.71)	0.002
Delayed diagnosis	1.08 (0.66–1.76)	0.756	1.40 (0.74–2.63)	0.303	0.68 (0.31–1.49)	0.331
Severe comorbidity (yes or no)	5.58 (4.64–7.21)	<0.001	4.57 (3.49–5.98)	<0.001	9.29 (6.19–13.95)	<0.001
**AKI stage at peak**						
Stage 1	Reference		Reference		Reference	
Stage 2	2.37 (1.81–3.10)	<0.001	2.60 (1.87–3.61)	<0.001	2.00 (1.24–3.24)	0.005
Stage 3	3.52 (2.65–4.68)	<0.001	4.07 (2.88–5.74)	<0.001	2.55 (1.49–4.37)	0.001
**Geographical region**						
North	Reference		Reference		Reference	
Southeast	0.53 (0.39–0.71)	<0.001	0.50 (0.34–0.72)	<0.001	0.58 (0.33–1.04)	0.069
Northwest	1.84 (1.24–2.74)	0.002	1.62 (0.99–2.65)	0.053	2.47 (1.22–4.99)	0.012
Southwest	0.95 (0.58–1.54)	0.824	0.84 (0.47–1.51)	0.569	1.26 (0.50–3.21)	0.628
RRT indication (yes or no)	1.84 (1.33–2.54)	<0.001	2.22 (1.49–3.33)	<0.001	1.20 (0.67–2.15)	0.541
Renal referral (yes or no)	0.58 (0.42–0.80)	<0.001	0.50 (0.33–0.74)	0.001	0.77 (0.43–1.38)	0.376
Academic vs local hospital	1.28 (0.97–1.68)	0.081	1.37 (0.99–1.89)	0.060	1.32 (0.76–2.27)	0.326
**GDP per head**						
Tertile 1	Reference		Reference		Reference	
Tertile 2	0.64 (0.42–0.96)	0.030	0.66 (0.40–1.09)	0.105	0.66 (0.31–1.43)	0.296
Tertile 3	1.40 (0.97–2.04)	0.075	1.29 (0.83–2.02)	0.258	1.69 (0.83–3.45)	0.149

## Discussion

Major, non-cardiovascular surgery was associated with an overall mortality of 5%^13^, but the crude mortality was significantly increased to 40–60% among patients who developed AKI^14–16^. In the last decade, the adoption of consensus recommended AKI definitions (including Risk Injury Failure Loss End-Stage Renal Disease [RIFLE], Acute Kidney Injury Network [AKIN], and Kidney Disease Improving Global Outcomes [KIDGO]) has allowed studies using standardized AKI-diagnostic methodology to assess the incidence of AKI and its implications post-operation, and consequently to provide a synthesis of evidence on treatment options, the prevention of AKI and improving patient outcomes^17–19^. However, systemic reports on post-operation AKI in non-cardiovascular surgical settings are limited worldwide compared to cardiovascular surgical settings^20–22^.

To overcome the potential obstacles of inadequate serum creatinine assays and overlooked AKI by doctors, we developed expanded diagnostic criteria for AKI, which include a decrease in the serum creatinine level and an extension of the interval between two serum creatinine assays. This method enabled us to record the incidence of AKI as accurately as possible^5^. Our survey suggests that the detection rate of AKI post-operation among all adult hospitalizations was 0.44%, including all patients undergoing operations; moreover, 47% of AKI cases developed AKI after surgery. This result is consistent with other published multicentre surveys on AKI from seven large cities in mainland China. One study reported a detection rate of AKI (according to KDIGO criteria) of approximately 2.3% in adult hospitalized patients^23^. The incidence rates reported in hospitalized patients are much lower than those reported from developed countries^17,24,25^. The Veterans Administration study and another multinational multicentre study showed that the incidence of AKI is generally 5–5.7% in all acute care hospitalizations, and it accounts for up to 20% of admissions to intensive care units (ICUs); of all AKI cases during hospitalization, approximately 30–40% occur in operative settings^15,26^. One possible explanation is that the present method of detecting AKI is mainly based on changes in serum creatinine; nevertheless, most inpatients did not have their serum creatinine repeatedly measured during their hospital stay. Therefore, the prevalence of AKI post-operation in China may be significantly underestimated throughout the country.

In the present survey, we focused on the incidence and mortality of AKI after non-cardiovascular operation. To achieve this, we excluded some operations that have specific additional aetiologies for AKI beyond those of major surgery in general, such as abdominal aortic surgery (ischaemia and cholesterol emboli), angiography and/or stenting procedure (contrast) and urological surgery (obstruction and urosepsis). Compared to non-surgical AKI, the patients with post-operation AKI had less history of CVD, hypertension, diabetes mellitus, and chronic kidney disease. Although AKI due to decreased kidney perfusion was the main cause of post-operation AKI (60.8%) in our survey, the second main cause was intrinsic renal AKI (24%). Given the higher rate of nephrotoxic drug exposure, such as aminoglycosides, vancomycin, antiviral drugs, and the higher rate of sepsis in patients with AKI after operation, the diagnosis of intrinsic renal AKI requires pathology; however, it is harder for patients undergoing a major operation to tolerate a renal biopsy. Moreover, in the present study, compared to non-surgical AKI patients, in-hospital mortality was lower in post-operative AKI patients (21.7% vs. 17.4%, respectively). However, there were no significant differences in recurring AKI in hospital and the proportion of patients who remained on RRT treatment. Even post-operative AKI could obviously extend the length of hospitalization [19 (10–32) vs. 22 (13–36) days]. Our results strongly indicate that there were no differences in aetiology between post-operative AKI and non-operative AKI. Conversely, more attention should be paid to the occurrence of potential intrinsic renal AKI in surgical patients. Unfortunately, until recently, there have been no effective drugs to prevent nephrotoxicity or infection induced post-operative AKI, such as high-dose atorvastatin^27^ or sodium bicarbonate^28^. Therefore, tightly controlled AKI occurrence, particularly in patients exposed to nephrotoxic factors or with comorbidities, is essential to improve the prognosis of patients undergoing a major operation.

We next investigated the epidemiology of post-operation AKI in each region. According to the present study, patients in the northwest had a lower rate of nephrotoxic drugs but higher rates of pre-renal AKI, more stage 3 AKI, and a higher in-hospital mortality. Additionally, the northwest had a higher non-recognition rate of AKI and a lower immediate diagnosis rate than the rest of regions of China. These results might have occurred because northwest China has a typical continental climate and a relatively low gross domestic product per head. Therefore, nephrologists in northwest China must pay increased attention to post-operation AKI through the education, training and development of an AKI alarm system that can improve the detection, diagnosis, and management of AKI.

A meta-analysis published in 2015 on the incidence and associations of AKI after major abdominal surgery reported that the pooled relative risk for hospital or 30-day mortality for patients with AKI was 12.6 (95% CI 6.8–23.4) compared to patients without AKI^6^. Thus far, there are limited data on mortality from post-operation AKI. In our study, the all-cause in-hospital mortality was 17.0% among patients with AKI after major surgery. The multiple regressions analysis showed that older age, more severe comorbidities, and a more severe peak AKI stage were common independent risk factors for in-hospital mortality in both surgical and non-surgical AKI patients. Unlike non-surgical AKI, RRT indication, renal referral and GDP per head were not associated with the risk of in-hospital mortality. Notably, CVD-basis was an independent risk factor for in-hospital mortality in surgical AKI patients. These results revealed that poor cardiovascular reserves could be associated with mortality risk induced by AKI^29^. In fact, after a non-cardiac operation, major cardiac complications are common; however, the capacity to predict these events is limited worldwide. The identification of pre-operative heart function enables doctors to determine the type of surgery or whether to proceed with intervention or manage conservatively. Recently, a prospective cohort study (Coronary CTA VISION) reported that coronary computed tomographic angiography could improve the estimation of risk for patients who experience perioperative cardiovascular death or myocardial infarction, although it might inappropriately overestimate the risk among patients who will not experience these outcomes^30^. Similarly, our results strongly support the need for a perioperative period risk assessment, particularly of heart function, to strengthen the early recognition of AKI to improve outcomes. Although the under-recognition of patients was an independent protective factor in the multivariate analysis, there was a possible explanation for this result: patients who were younger, had a milder AKI stage, and non-CKD basis were more prone to misdiagnosis or delayed diagnosis of AKI after operation.

To our knowledge, a multicentre study of AKI in non-cardiovascular operations with a sample of this size is unprecedented. We conducted a thorough investigation of AKI in non-cardiovascular operations in China and obtained sufficient information from stratified analyses of regions and economic development. This information will allow national health authorities to identify and address factors associated with post-operation AKI in different regions. We first compared the differences in clinical features, aetiology and risk factors of in-hospital mortality between operation- and non-operation-related AKI. Because determining the cause of operative AKI is almost impossible when solely relying on kidney pathological diagnosis, such comparisons could provide more reference for clinical diagnosis and treatment.

However, our study also has limitations that should be considered. First, this retrospective study depended on repeated Scr tests that were independent of urinary output to screen for AKI cases. Hence, patients with low urine output but stable Scr levels could be missed. Therefore, the actual misdiagnosis of AKI might be higher than our current report and our previous report. Second, similar to our previous reports, the patients with more severe conditions tended to get more attention; thus, milder cases may have been missed. Third, this survey selected 2 months, January and July, to be representative of winter and summer in mainland China; however, this sample method might affect operative quantity. Fourth, when designing the nationwide survey, the patients who developed post-operation AKI were enrolled in the next analysis, and the patients who did not develop AKI were not observed. Therefore, we calculated the incidence and mortality rates based on all of the in-hospital AKI patients but not the in-hospital surgical patients. In addition, we investigated a limited number of potential nephrotoxic drug exposures, and we did not investigate the role of some protective drugs on AKI. Despite these limitations, our survey provides critical and novel insights into the epidemiology and major risk factors of post-operation AKI in China.

In summary, AKI after a non-cardiovascular operation has become a huge medical burden in China. The features of AKI after a non-cardiovascular operation varied substantially in different regions of China. There were no differences in aetiology between post-operative AKI and non-operative AKI. In particular, patients exposed to nephrotoxic factors or comorbidities require more attention to monitor for the potential occurrence of intrinsic renal AKI. Older age, more severe comorbidities, a history of CVD, a more severe peak AKI stage, and being in the northwest region of China were significantly associated with increased in-hospital mortality risk in post-operation AKI patients. A perioperative period risk assessment is necessary to strengthen the early recognition of AKI and improve outcomes.

## References

[1] Mehta RL (2015). International Society of Nephrology’s 0by25 initiative for acute kidney injury (zero preventable deaths by 2025): a human rights case for nephrology. Lancet.

[2] Coca SG, Yusuf B, Shlipak MG, Garg AX, Parikh CR (2009). Long-term risk of mortality and other adverse outcomes after acute kidney injury: a systematic review and meta-analysis. Am J Kidney Dis.

[3] Susantitaphong P (2013). World incidence of AKI: a meta-analysis. Clin J Am Soc Nephrol.

[4] Remuzzi G, Horton R (2013). Acute renal failure: an unacceptable death sentence globally. Lancet.

[5] Yang L (2015). Acute kidney injury in China: a cross-sectional survey. Lancet.

[6] O’Connor ME, Kirwan CJ, Pearse RM, Prowle JR (2016). Incidence and associations of acute kidney injury after major abdominal surgery. Intensive Care Med.

[7] Bihorac A (2009). Long-term risk of mortality and acute kidney injury during hospitalization after major surgery. Ann Surg.

[8] Kheterpal S (2007). Predictors of postoperative acute renal failure after noncardiac surgery in patients with previously normal renal function. Anesthesiology.

[9] Hansen MK (2013). Post-operative acute kidney injury and five-year risk of death, myocardial infarction, and stroke among elective cardiac surgical patients: a cohort study. Crit Care.

[10] Bell S (2014). Risk of AKI with gentamicin as surgical prophylaxis. J Am Soc Nephrol.

[11] Grams ME (2016). Acute Kidney Injury After Major Surgery: A Retrospective Analysis of Veterans Health Administration Data. Am J Kidney Dis.

[12] Kidney DI (2012). G.O.A.K.I.W.G. KDIGO Clinical practice guideline for acute kidney injury. Kidney Int Suppl.

[13] Pearse RM (2012). Mortality after surgery in Europe: a 7 day cohort study. Lancet.

[14] Chertow GM, Burdick E, Honour M, Bonventre JV, Bates DW (2005). Acute kidney injury, mortality, length of stay, and costs in hospitalized patients. J Am Soc Nephrol.

[15] Uchino S (2005). Acute renal failure in critically ill patients: a multinational, multicenter study. JAMA.

[16] Hoste EA (2015). Epidemiology of acute kidney injury in critically ill patients: the multinational AKI-EPI study. Intensive Care Med.

[17] Uchino S, Bellomo R, Goldsmith D, Bates S, Ronco C (2006). An assessment of the RIFLE criteria for acute renal failure in hospitalized patients. Crit Care Med.

[18] Section 2: AKI Definition. *Kidney Int Suppl* (2011) **2**, 19–36 (2012). 10.1038/kisup.2011.32 PMC4089595 25018918

[19] Bellomo R, Ronco C, Kellum JA, Mehta RL, Palevsky P (2004). Acute renal failure - definition, outcome measures, animal models, fluid therapy and information technology needs: the Second International Consensus Conference of the Acute Dialysis Quality Initiative (ADQI) Group. Crit Care.

[20] Thakar CV (2013). Perioperative acute kidney injury. Adv Chronic Kidney Dis.

[21] Demirjian S (2012). Predictive models for acute kidney injury following cardiac surgery. Am J Kidney Dis.

[22] Huen SC, Parikh CR (2012). Predicting acute kidney injury after cardiac surgery: a systematic review. Ann Thorac Surg.

[23] Xu X (2015). Epidemiology and Clinical Correlates of AKI in Chinese Hospitalized Adults. Clin J Am Soc Nephrol.

[24] Thomas M, Sitch A, Dowswell G (2011). The initial development and assessment of an automatic alert warning of acute kidney injury. Nephrol Dial Transplant.

[25] Srisawat N, Kellum JA (2011). Acute kidney injury: definition, epidemiology, and outcome. Curr Opin Crit Care.

[26] Thakar CV, Christianson A, Freyberg R, Almenoff P (2009). & Render, M. L. Incidence and outcomes of acute kidney injury in intensive care units: a Veterans Administration study. Crit Care Med.

[27] Billings FT (2016). High-Dose Perioperative Atorvastatin and Acute Kidney Injury Following Cardiac Surgery: A Randomized Clinical Trial. JAMA.

[28] Cho JS (2017). Effect of perioperative sodium bicarbonate administration on renal function following cardiac surgery for infective endocarditis: a randomized, placebo-controlled trial. Crit Care.

[29] Saratzis A, Shakespeare J, Jones O, Bown MJ, Mahmood A (2017). & CHE, I. Pre-operative Functional Cardiovascular Reserve Is Associated with Acute Kidney Injury after Intervention. Eur J Vasc Endovasc Surg.

[30] Sheth T (2015). Prognostic capabilities of coronary computed tomographic angiography before non-cardiac surgery: prospective cohort study. BMJ.

